# Lectures on the Diseases of Infancy and Childhood

**Published:** 1850-02

**Authors:** 


ART. IV.—*Lectures on the Diseases of Infancy and Childhood.* By CHARLES WEST, M. D., F. R. C.; Physician to the Royal Infirmary for Children; Physician, Accoucheur to the Middlesex Hospital; and Lecturer on Midwifery at St. Bartholomew's Hospital. 1 vol., 8vo., pp. 451. Philadelphia: Lea & Blanchard. 1850.

*On the Diseases of Infants and Children.* By FLEETWOOD CHURCHILL, M. D., M. R. I. A., Hon. Fellow of the College of Physicians, Ireland; Hon. Member of the Philadelphia Medical Society, etc., etc., author of "The Theory and Practice of Midwifery," "On the Diseases of Females," etc., etc. pp. 636. Lea & Blanchard. 1850.

*A Practical Treatise on the Diseases of Children.* By D. FRANCIS CONDIE, M. D., Sec. of the College of Physicians; Member of the American Medical Association, &c., &c. Third edition, revised and augmented. pp. 703. Philadelphia: Lea & Blanchard. 1850.

No better evidence could be given of the increasing attention that is paid to the diseases of children, by medical observers, than the appearance of three such works, as we have named at the head of this article, devoted exclusively to the diseases of early life. Nearly all other departments of practical medicine have received a due amount of contribution from authors; the diseases of children have been, to a considerable extent neglected. Maunsell and Evanson, Eberle and Dewees published works on the subject, but they neither commanded nor deserved the confidence of the profession. They bear on their pages, the indelible marks of the mere book maker, and are of but little use in the practice of medicine. They claim but little of a philosophical spirit, and possess no more than they claim.

The works before us are of a different order—each of them is a valuable contribution to medical literature, and deserves the attention of medical men. The principles inculcated in these volumes are based upon the present improved condition of medical science, and are the result of close and discriminating observation in widely extended fields for the acquisition of facts, and the observance of the phenomena of disease. Professor West informs us that his observations are founded upon nearly fourteen thousand cases; Dr. Churchill's opportunities have been great, and have been well improved, and Dr. Condie's admirable chapters show that he has been a close, vigilant and intelligent watcher of the diseased actions of childhood. In many respects we deem Dr. Condie's work the best of the three, for American practitioners. It is unnecessary for us to dwell upon the modifying influences exerted upon disease, by climate, habits of life, character of parents, modes of dress, the nature of food, and the condition of habitations. These circumstances are familiar to all well informed medical men, and should be correctly appreciated by all who attempt the practice of medicine. If we take the principles on blood-letting, educed by Dr. Marshall Hall from observations among the population of Manchester, and apply them to any population in the United States, we shall fall into deplorable and disastrous practices. In the London Hospitals erysipelas generally demands a stimulant treatment; in this country such treatment would be attended with serious consequences. Examples of a similar character crowd upon us, but it is unnecessary to dwell upon them. The fact, we are endeavoring to inculcate, is too palpable to need an extended illustration. Principles are invaluable, and always deserve attention; but we cannot be too careful in submitting the judgement to the rules that have been formed in, and that are adapted to a different state of things from what is found among the sick in this country. A child in one of the Western schools, on being called upon to define the difference between a principle and a rule, said a rule is the guide post that points out the road to the traveler, a principle, not only shows the road, but accompanies the traveler to his point of destination. The idea, contained in this lucid definition cannot be too forcibly impressed upon the attention of medical readers. The man who follows rules merely, degenerates into the practices of the routinist, and from that sinks into something worse. But he who accumulates principles, continually rises towards the most elevated regions of a noble art, and makes himself a blessing to humanity.

For the reasons we have given, we prefer the new edition of Dr. Condie's work on the Diseases of Children, to either of the others, but we must not be understood as disparaging the treaties of Drs. West and Churchill. They are valuable in their places; they deserve to be studied with care, and they command respect by the richness, plenitude and variety of their facts. What we have said on the subject of the superiority of Dr. Condie's work was said, in order to impress upon the reader the necessity of inquiring in what fields of observation a medical writer labored, because that inquiry is important in making up a judgement upon the character of the principles and rules that are inculcated. In London Dr. West's work would be more useful than Dr. Churchill's, and the work of the latter would be more useful in Dublin, than that of the former, and, by parity of reasoning Dr. Condie's would be more valuable in Philadelphia than either of the others.

We have examined these works carefully, and take pleasure in bearing testimony to their value. We feel no kind of disposition to use the critic's rod upon works that have afforded us more than ordinary gratification, and which are calculated to exert a beneficial influence upon the practice of medical readers. As an illustration of their value, when compared with the works of Maunsell and Evanson, Dewees and Eberle, we ask the reader to examine the subject of gangrenopsis in each of the works named at the head of this article, and then let him inquire of the other authors what they have to say upon this frequent, distressing, deforming and fatal disease. Why it has entirely escaped the attention of many writers on the diseases of infancy we are at a loss to determine, for it occurs often enough to be a serious evil, and is fatal enough to arouse the most lethargic physician. The first case of gangrenopsis we ever met impressed itself too forcibly on our memory ever to be forgotten. A beautiful little girl was the victim, and the general impression of those who saw the child was that she was a martyr to salivation. We knew that it was impossible that the phagedenic ulceration could be the result of mercury, for the child had taken nothing of the kind for three weeks before the gangrenopsis showed itself, and the destructive peculiarities of that affection soon became so manifest, that all doubts on the subject were dispelled. The case terminated fatally. Within the past few weeks, we have seen another, in which there could have been nothing like salivation. Gangrenopsis, in this case, followed a severe attack of measles in a young subject. Ulceration commenced in the inside of the cheek, and rapidly traveled along the cheek and gums. The teeth were loosened by the gangrene, and were extracted by the child. The gangrenous ulcer afterwards attacked the left nostril, and speedily terminated the child's sufferings in death. All the treatment recommended by the authors was diligently pursued; more faithful nursing never was given, but despite of all remedies, most unremittingly applied, the disease traveled to its fatal issue with an unfaltering step. We have seen diseases of a most formidable character, but we know of none that we would not as lief meet as gangrenopsis. There seems to be but little pain attending its devastating progress; it sometimes sweeps along its fatal course with surprising rapidity, and at others pursues an intermitting career, pausing long enough to flatter the physician and nurses into hope, to be dispelled in the midst of an apparent fruition, and closing the scene in death, with as much certainty as the cases that proceed with greater celerity.

We are glad that the treatises before us have recognised, and given deserved prominence to this distressing affection. Each of the works gives a faithful portrait of the disease, and we commend our fellow-laborers in practice to the study of the chapters devoted to gangrenopsis. Many an instance of the disease has passed for salivation, where there was no kind of connection with that untoward practice.

We refer to this particular case as an example of improvement in the recent treatises upon the diseases of infancy. A multitude of other examples will spring to the view of the diligent reader of the works of West, Churchill, and Condie. We commend these works to studious practitioners, and with the commendation we urge a careful observation of facts, on the part of physicians. No particular excellence can be attained by a blind devotion to the works of others; each man has an eye, a mind, and a judgement that were given to him for his own use, not to be manacled by others. Nature opens her broad fields to all; her sun lights up phenomena for the eye of all who are disposed to look at them, and he is unjust to himself, who neglects opportunities of daily improvement. The bed-side is the proper place for the study of diseased actions; the books should be used only as lamps that shed their light around the patient.

The book of Dr. Churchill is noticeable for one singular fact: it is the first instance we know of, where a British writer has sought an American publisher. The work of Dr. Churchill was written expressly for Lea & Blanchard, and is very appropriately dedicated to Drs. R. M. Huston, Isaac Hays, and George Shattuck, jr.

